# Synthesis of a novel magnetic nanomaterial for the development of a multielemental speciation method of lead, mercury, and vanadium via HPLC-ICP MS

**DOI:** 10.1007/s00604-023-05877-x

**Published:** 2023-07-17

**Authors:** Pablo Montoro-Leal, Juan Carlos García-Mesa, Irene Morales-Benítez, Laura Vázquez-Palomo, María del Mar López Guerrero, Elisa I. Vereda Alonso

**Affiliations:** Department of Analytical Chemistry, Faculty of Sciences, University of Malaga, 29071 Málaga, Spain

**Keywords:** Magnetic graphene oxide, Multielemental speciation, Solid-phase extraction, HPLC-ICP MS

## Abstract

A new magnetic functionalized material based on graphene oxide magnetic nanoparticles named by us, M@GO-TS, was designed and characterized in order to develop a magnetic solid-phase extraction method (MSPE) to enrich inorganic and organic species of lead, mercury, and vanadium. A flow injection (FI) system was used to preconcentrate the metallic and organometallic species simultaneously, while the ultra-trace separation and determination of the selected species were achieved by high-performance liquid chromatography coupled to inductively coupled plasma mass spectrometry (HPLC-ICP MS). Therefore, preconcentration and separation/determination processes were automated and conducted separately. To the best of our knowledge, this is the first method combining an online MSPE and HPLC-ICP MS for multielemental speciation. Under the optimized conditions, the enrichment factor obtained for Pb^II^, trimethyllead (TML), Hg^II^, methylmercury (MetHg), and V^V^ was 27. The calculated LOD for all studied species were as follows: 5 ng L^−1^, 20 ng L^−1^, 2 ng L^−1^, 10 ng L^−1^, and 0.4 ng L^−1^, respectively. The RSD values calculated with a solution containing 0.5 μg L^−1^ of all species were between 2.5 and 4.5%. The developed method was validated by analyzing Certified Reference Materials TMDA 64.3 for total concentration and also by recovery analysis of the species in human urine from volunteers and a seawater sample collected in Málaga. The t statistical test showed no significant differences between the certified and found values for TMDA 64.3. All the recoveries obtained from spiked human urine and seawater samples were close to 100%. All samples were analyzed using external calibration. The developed method is sensitive and promising for routine monitoring of the selected species in environmental waters and biological samples.

**Graphical abstract:**

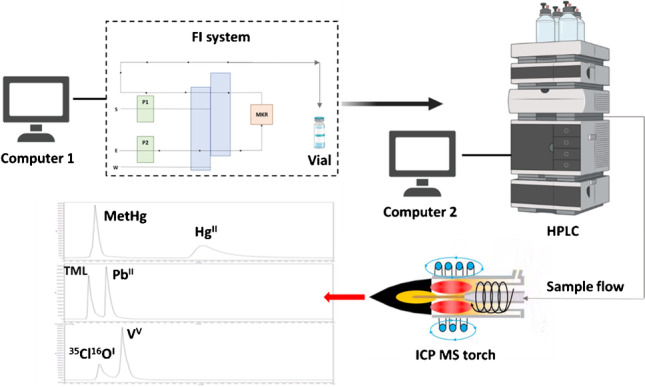

**Supplementary Information:**

The online version contains supplementary material available at 10.1007/s00604-023-05877-x.

## Introduction

Mercury and derivatives are considered one of the most toxic pollutants, which arise from natural and anthropogenic sources [1]. Serial damage is caused by human vital organs, including heart, brain, stomach, kidneys, and lungs, even at trace concentration [2]. Elemental (Hg^0^), inorganic (Hg^I^ and Hg^II^), and organic mercury species such as MetHg^I^ and ethylmercury (EtHg^I^) are the most common Hg species in the environment [3, 4], presenting different toxicity levels. The liposolubility, bioaccumulation, neurotoxicity, and transmission capacity of MeHg^I^ through the food chain makes this species the most dangerous chemical form of mercury [4]. Furthermore, inorganic mercury can be easily transformed into MeHg^I^ in the environment. The maximum concentration allowed of this element in surface water by the EU is 0.07 μg L^−1^ [5]. Besides, lead is one of the oldest known toxins and mainly arises from several anthropogenic sources such as paint, ceramic, and ink industries as well as the automobile batteries field [6]. Pb intoxication is well known, causing the reduction of the enzymatic activity in humans, developing diseases like anemia, nephritic colic, infertility, and neuromuscular difficulties [7, 8]. Moreover, Pb is accumulative [9], and exposure to low concentrations is related to neurological diseases, loss of hearing, and cancer development, among other dysfunctions [10]. The toxicity of lead also depends on the chemical form, being considered the organic species TML the most toxic form to mammals, which is accumulated in the brain and inhibits glucose [11]. World Health Organization (WHO) establishes a maximum concentration of total Pb in drinking water of 10 μg L^−1^ [12]. Vanadium exists in a variety of oxidation states from V^−I^ to V^V^. However, the most stable and the most extended species in the environment is V^V^, which shows toxicity in biological systems. Vanadium is also released into the environment in large amounts, mainly derived from the use of fossil fuels and other human activities [13, 14].

Considering all the information exposed, the development of simple and sensitive analytical speciation methods for the determination of traces and ultratraces of Pb, Hg, and V in environmental and biological samples is crucial [5, 15]. In order to develop a speciation method, the use of an atomic spectrometer coupled with a separation system is really extended. The combination of inductively coupled plasma mass spectrometry (ICP MS) and high-performance liquid chromatography (HPLC) has been previously proposed to achieve the separation and determination of a high variety of elements with high sensitivity and wide linear ranges, including mercury, lead, and vanadium species such as Pb^II^, TML, triethyllead (TEL), Hg^II^, MetHg, EtHg, V^V^, and V^IV^ [16–21]. However, despite the excellent characteristics of this technique, the mixture of the sample and the mobile phase during the HPLC separation generate an additional dilution. Consequently, the sensitivity of the method can be compromised for the monitoring of traces in samples with highly complex matrices such as environmental and biological samples [22]. Therefore, a preconcentration procedure prior to analysis is needed in order to ensure an adequate analysis.

GO is a graphite mono-layer with an extended π-π system that presents a high surface concentration of oxygen-containing functional groups, including epoxy, carboxylic acid, carbonyl, and hydroxyl groups. Consequently, GO can interact with a huge variety of analytes through physical, hydrogen bonds, and aromatic interactions. However, although the adsorption on GO is good, this material shows several limitations derived from its application, being tedious to use, slow separation from the matrix, and high sample volume requirements [23]. Besides, iron oxide MNPs (Fe_3_O_4_) have been exploited as solid-phase sorbents due to their degradability, biocompatibility, chemical stability, low toxicity, and high magnetic response. This last property allows a variation of the classical solid-phase extraction known as magnetic solid-phase extraction (MSPE) [24, 25]. MSPE procedures have gained great interest in recent years for speciation analysis due to the advantages previously mentioned. Among the potential magnetic nanomaterials selected to perform the MSPE process in the trace, and even ultra-trace element speciation analysis, the relevance of MGO is increasing. MGO has already been applied for the speciation of Al, Cr, As, Se, Ag, Cd, Hg, and Tl [23]. However, no analytical method has been found in the bibliography for Pb speciation. Besides, the majority of described methods are developed in in-batch mode, which makes their automatization impossible.

However, magnetic nanomaterials can be easily retained inside a microcolumn or a knotted reactor with the aid of an external magnetic field during the adsorption and elution processes. In this way, the MSPE can be achieved in a totally automatic mode simply by replacing the sample loop of an injection valve with the reactor filled with magnetic nanomaterial. In this study, a patented magnetic nanomaterial previously synthesized by P. Montoro-Leal et al. [26] based on a double coupling between GO and MNPs (M@GO) was used. The synthesis procedure provides a greater and better functionalization, where the chemical group is attached to both the MNPs and the GO layer. Then, M@GO was functionalized with the chelant group methylthiosalicilate (MTS) in order to increase the selectivity and the loading capacity of the material toward metal ions and their derivatives. The resulting new nanomaterial (M@GO-TS) was adequately synthesized and characterized. M@GO-TS was applied for the fabrication of a magnetic knotted reactor (MKR) to develop an online preconcentration system. The strategy of this method is based on the enrichment of Pb^II^, MetPb, Hg^II^, MetHg, and V^V^ prior to HPLC-ICP MS determination. The analytes were carefully selected, attending to their presence in aqueous samples and toxicity. The validation of the method was performed by analyzing a Certified Reference Material (Fortified lake water TMDA 64.3) for total Pb and also by recovery analysis of the species in human urine from volunteers and a seawater sample collected in Málaga. The resulting analytical method was proven to be efficient and reliable for the detection of the species in complex matrix samples, being adequate for routine monitoring of organic and inorganic species of Hg, Pb, and V in environmental waters and biological samples. The analytes (organic and inorganic species of highly toxic elements) can be simultaneously collected and preconcentrated in situ with the automatic MSPE procedure. Then, 1 mL of eluate can be carried to the laboratory for its injection in the optimized multielemental HPLC-ICP MS method. Thus, this methodology could provide an automatic control system for the seas and oceans with continuous sampling and pretreatment of the samples prior to the speciation analysis in the laboratory. To the best of our knowledge, this is the first method based on an automatic MSPE process combined with HPLC-ICP MS for multielemental determination of organic and inorganic species of mercury, lead, and V [23].

## Experimental

### Instrumentation and materials

An HPLC Perkin Elmer Flexar was connected to an ICP MS Perkin Elmer NexION 2000 (Waltham, MA, USA). For HPLC separation of the selected species, a Kinetex 5 μm EVO C18 column 4.6 × 250 mm 100 Å from Phenomenex (Torrance, CA, USA) was applied. The HPLC presents an autosampler module with a 100-position tray for the automation of the injection of the chromatographic vial content. HPLC-ICP MS system is controlled by Empower software. The ICP MS instrument was used with standard nickel sampler and skimmer cones, which was daily optimized and operated as recommended by the manufacturer. One of the most important parameters to be optimized is the nebulizer gas flow rate, being adjusted with the following criteria: Ce^II^ (70)/Ce (140) ratio and CeO (156)/Ce (140) must be less or equal to 0.03 and 0.025, respectively. An 80-cm 0.8 mm i.d. capillary tube was used to connect the outlet of the HPLC column and the ICP MS nebulizer. The optimum operating conditions of the HPLC-ICP MS system are shown in Table 1. In order to preconcentrate the species prior to analysis, a flow injection (FI) system PerkinElmer FIAS-400 with two peristaltic pumps and a five-port way rotary valve was used.

**Table 1:** HPLC-ICP MS conditions

ICP MS	
Monitored signals	^208^Pb, ^202^Hg, ^51^V
Replicates	3
Radiofrequency power, W	1600
Waste flow rate, mL min^−1^	10
Distance from HPLC to ICP MS, cm	80
Dwell time, ms	2000
Nebulizer type	Cyclonic chamber
Gas flows, L min^−1^ (plasma, auxiliary, nebulizer)	15/1.2/1.0
Torch alignment, mm (horizontal, vertical, depth)	0.56/0.59/0.00
HPLC	
Injection volume, μL	100
Column temperature, °C	20±1
Mobile phase flow rate, mL min^−1^	1.3
Medium pressure (psi)	2200
Column	Kinetex 5 μm EVO C18 4.6 × 250 mm
Mobile phase A	Eluent: 7 mM thiourea + 40 mM H_3_PO_4_
Mobile phase B	0.16 mM TBAOH in H_3_PO_4_ pH 4.5
Chromatographic program	100.0%A, 0.0%B 0.1 min (initial) 0.0%A, 100.0%B 1.5 min (ramp of 1.2% s^−1^) 0.0%A, 100.0%B 18.4 min

For the characterization of M@GO-TS, several instruments were used: A Spectrum 100 FT-IR spectrometer was acquired from Perkin Elmer (Shelton, CT, USA) to obtain FT-IR spectra. The samples were measured using KBr pellets, in which the concentrations for the samples were 0.5% (wt/wt) approximately. XPS analysis was performed with a Physical Electronics ESCA 5701 instrument (Chanhassen, MN, USA). The binding energies (BE) were observed, considering the position of the C 1s peak at 284.8 eV as a reference. The residual pressure in the analysis chamber was maintained below 3 × 10^−9^ torr during data acquisition. The microstructure of M@GO-TS was observed and studied by TEM images using a JEOL JEM-1400 (Peabody, MA, USA) and N_2_ adsorption isotherms registered with Micromeritics ASAP 2020 V4.02 (Norcross, GA, USA). The composition of the materials (C, N, O, S) was studied by CHNOS elemental analysis from LECO TruSpec Micro CHNSO (St. Joseph, MI, USA). The adsorption capacity of the prepared material was determined with a vortex mixer VX-2500 Multi-Tube Journal Pre-proof from VWR international (Radnor, PA, USA) and ICP-MS PerkinElmer Nexion 2000. Finally, in order to study the magnetic properties of M@GO-TS, a Vibrating-sample Magnetometer from NanoScale Biomagnetics (Zaragoza, Spain) was used.

MKR has been previously used by the research group as the best solution to pack magnetic materials [27], being fabricated as follows: 50 mg of M@GO-TS was introduced in a PTFE tube (500 mm, 0.5 mm i.d.) knotted around a circular Nd/Fe/B magnet (outer diameter: 40 mm; inner diameter: 23 mm; height: 5 mm; holding strength: 81.4 N) and sandwiched between other two identical circular magnets. At both ends of the PTFE tubes, polyethylene filters (Omnifit, Cambridge, UK) were fixed to prevent material loss. The MKR was placed in the sample loop of the five-port rotary valve of FIAS-400AS, which was not connected directly to NexION 2000. The FI system was controlled by a second independent computer using Perkin Elmer Syngistix software, and the outlet tube was collected into the chromatographic vial.

### Reagents, standards, and solutions

Doubly de-ionized water (18 MΩ cm) was used throughout. High-purity reagents were used in all experiments, and all plastic and glassware were cleaned with 10% w/w nitric acid and stored soaked with this acid. They were rinsed several times with doubly de-ionized water immediately before use. In this work, 65% HNO_3_, ≥99.8% acetic acid, 99% sodium acetate, ≥99.5% boric acid, and ≥98% sodium tetraborate were used to prepare buffer solutions. Commercial 1000 mg L^−1^ solutions of Pb^II^, Hg^II^, and V^V^ were used for the preparation of standards. 85% ortho-phosphoric acid, ≥98.5% L-cysteine, ≥99% ethylenediaminetetraacetic acid, ≥99% thiourea, and 40% tetrabutylammonium hydroxide (TBAOH) were used for mobile phase and eluent preparation. For the synthesis of M@GO, graphite powder, sodium chloroacetate, ferrous chloride tetrahydrate (FeCl_2_·4H_2_O), ferric chloride hexahydrate (FeCl_3_·6H_2_O), 37% HCl (wt/wt), ammonium hydroxide 30% (wt/wt), 97% 3-aminopropyltriethoxysilane, ≥99% tetraethoxysilane (TEOS), methanol, sodium chloride, ≥99.5% ethylenediamine (EDA), and 99% N,N’-dicyclohexylcarbodiimide (DCC). 97% methyl thiosalicylate (MTS) was used for functionalization. All reagents were purchased from Merck (Darmstadt, Germany), except for 1000 mg L^−1^ methylmercury chloride (MetHg) from Alfa Aesar (Ward Hill, MA, USA) and >99% trimethyllead chloride from LGC Standards (Barcelona, Spain).

The certified reference material analyzed to determine the accuracy of the proposed procedure was obtained from the National Research Council of Canada (NRCC): Fortified Lake Water TMDA 64.3. In order to study the applicability of the method, seawater and urine samples were collected in polypropylene bottles (previously cleaned by soaking for 24 h in 10% (w/w) nitric acid and finally rinsed thoroughly with de-ionized water before use). Samples were immediately filtered by using a membrane of 0.45-μm pore size cellulose nitrate filters from Millipore (Bedford, MA, USA). After that, the samples were acidified to 0.1% v/v by the addition of concentrated HNO_3_ and were stored in low-density polypropylene bottles at 4 °C for less than 3 days until analysis.

### Synthesis of M@GO-TS

The exfoliation process described by Diagboya et al. was used to prepare GO from natural graphite, being purified by several centrifugation cycles [28]. The synthesis of silica-coating MNPs was optimized by the research group [29]. Both materials were coupled for the preparation of M@GO, described as method 3 by P. Montoro-Leal et al. [30]. In this work, the organic group MTS was coupled on the material surface to obtain M@GO-TS in order to increase the loading capacity and selectivity toward metal ions and their derivatives. This is the first time M@GO-TS has been used to develop an analytical method. The Supplementary information (SM) shows the functionalization process and the structure of the resulting material (Supplementary Figs. SM1 and SM2). M@GO-TS was adequately synthesized and characterized by using TEM, nitrogen adsorption isotherms, FT-IR, elemental analysis, XPS, and VSM.

### Study of the adsorption capacities toward metal ions and derivatives

For the study of the capacity of adsorption, the suspensions were prepared by mixing 5 mg of the magnetic material and 25 mL, 2 mg L^−1^ of V, Cr, Mn, Co, Cu, As, Cd, Sb, Hg, Pb, MetHg, and TML aqueous solution. The metals were separated into three groups to avoid the saturation of M@GO-TS and the incompatibility of the ICP MS to distinguish the different chemical forms of an element: As, Hg, Pb, Sb, and Mn (group 1); Cr, Cu, Cd, Co, and V (group 2); MetHg and TML (group 3). The different groups were measured separately at acid and basic pH (1, 5, and 8). The pH was adjusted by using nitric acid, acetate/acetic acid buffer solution, and borax/boric acid buffer solution, respectively. The suspensions were vortexed for 10 min, and the materials were separated from the suspension with the aid of a magnetic field. The supernatant solutions were analyzed by ICP MS, and the adsorption capacities were calculated by difference with the initial concentration. The maximum capacity of adsorption that could be obtained was 10 mg g^−1^ material. The same experimental procedure was applied using alternative materials previously synthesized by the research group [30], M@GO-DPTH and M@GO-PSTH, functionalized with [1,5-bis (di-2-pyridyl) methylene] thiocarbonohydrazide, and [1,5-bis(2-pyridyl)3-sulfophenylmethylene] thiocarbonohydrazide, respectively. The adsorption capacity of these materials toward Pb^II^ and Hg^II^ was studied at pH 5 and compared with the results obtained with M@GO-TS.

### Preconcentration procedure and measurement

**Fig. 1:**
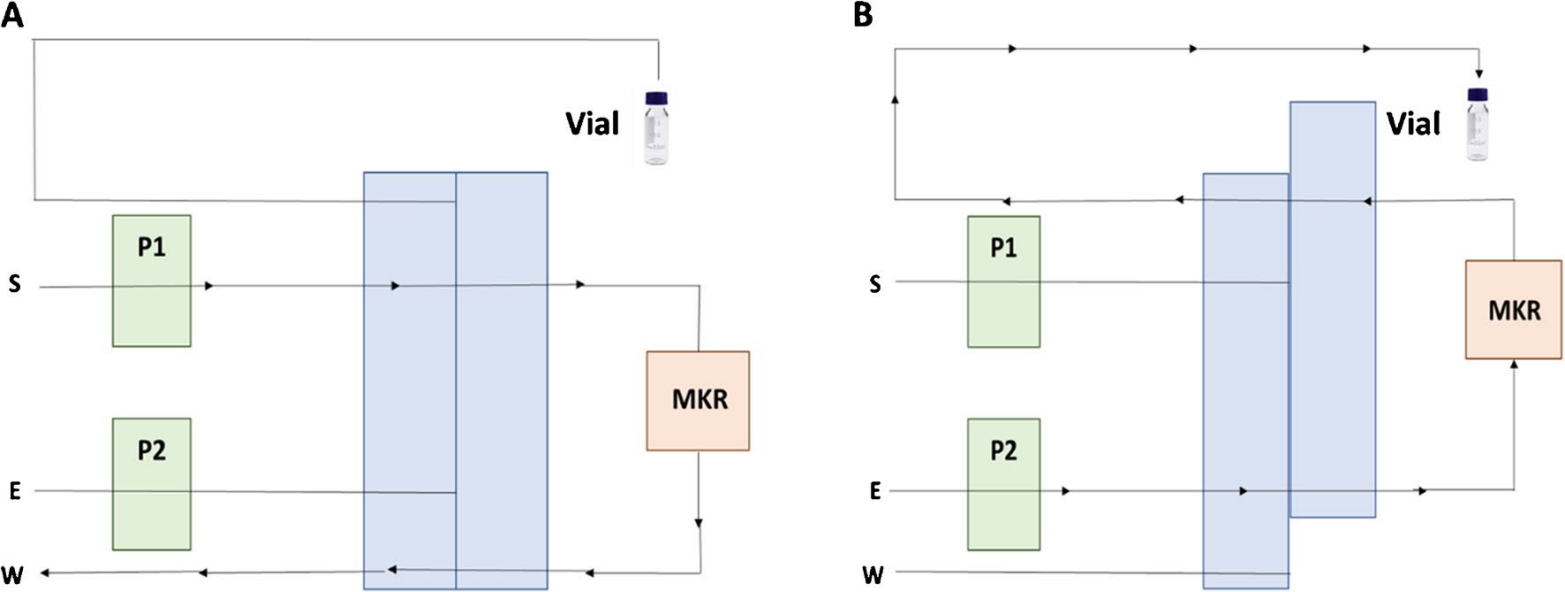
Scheme of the FI system used for preconcentration: (**A**) loading step and (**B**) elution step. Peristaltic pumps (P1 and P2), magnetic knotted reactor (MKR), waste (W), sample (S), and eluent (E) [22]

**Fig. 2:**
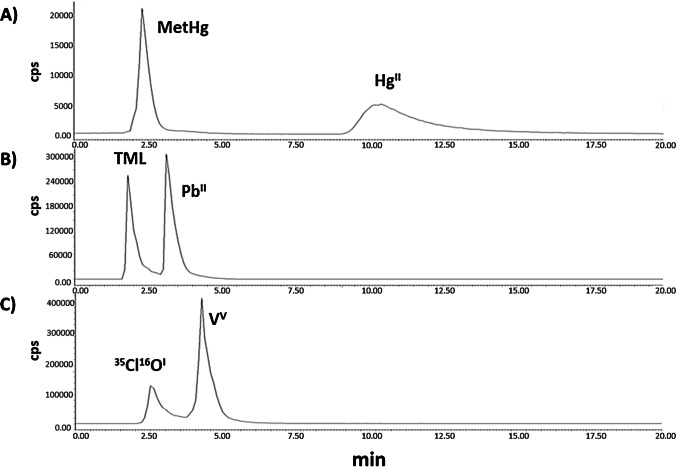
Chromatogram of the selected species under optimum conditions. *Y*-axis signal intensity (c/s) and *X*-axis time (min). Total time of the chromatogram: 20 min. Monitored signals: (**A**) Hg^202^, (**B**) Pb^208^, and (**C**) V^51^

The scheme of the FI system used for preconcentration is shown in Fig. 1. The sample loop of the injection valve of the FI system used for online automatic preconcentration was replaced by the MKR filled with 50 mg of M@GO-TS. The sample adjusted at pH 3.5 (10% acetate/acetic acid buffer solution) was loaded with the valve in position 1, being pumped via P1 through the MKR for 10 min at 2.7 mL min^−1^. During the loading step, the target analytes (Pb^II^, TML, Hg^II^, MetHg, and V^V^) were retained in MKR, while the other sample matrix components were eliminated. Then, the sample pump P1 stopped, and the valve position changed to position 2. During the elution step, the eluent (7 mM thiourea + 40 mM H_3_PO_4_) was passed through the reactor, and the analytes were extracted at 1.5 mL min^−1^. At the end of the system tube, a chromatographic vial was used to collect 1 mL of the eluent containing the preconcentrated analytes. It has been estimated that the eluent solution containing the target analytes is stable for at least 24 h at 5 °C in the vial. Finally, 100 μL of the vial content was injected in the HPLC-ICP MS system for the measurement, and the species were separated in a single run (Fig. 2); 12 min was considered enough for the monitorization of all species, and the retention times for Pb^II^, TML, Hg^II^, MetHg, and V^V^ under the optimum conditions were 3.2, 1.9, 10.2, 2.3, and 4.3 min, respectively. An additional peak was observed at 2.3 min when V^51^ was monitored. According to the bibliography, this signal was assigned to the interference ^35^Cl^16^O^I^ [21]. This semiautomatic system (online MSPE + HPLC-ICP MS) was initially proposed by the research group and applied by P. Montoro-Leal et al. for the speciation analysis of arsenic species [22]. The optimized chromatographic program and HPLC-ICP MS conditions were summarized in Table 1. Two different solutions were involved in the chromatographic separation (eluent as phase A and 0.15 mM TBAOH in H_3_PO_4_ pH 4.5 as phase B). In order to ensure the reproducibility of the method, an initial gradient was included. This gradient allows more efficient control of the pH solution when the sample is injected in the HPLC-ICP MS system from phase A (pH = 1.5) to phase B (pH = 4.5), being gradually modified in 1.5 min. For validation, the certified and real samples were measured following the procedure explained before. The aliquot of the sample (0.5–20 mL) was introduced in 100-mL volumetric flasks. Then, the pH was adjusted using a 10% buffer solution, and doubly de-ionized water was added up to the mark.

### Optimization strategy

In this work, two groups of parameters can be considered to be optimized: (1) parameters related to online MSPE, including eluent composition, sample flow rate, and elution flow rate, and (2) separation system parameters. Two optimization strategies were followed: univariate, by means of changing one parameter and keeping the others constant, and multivariate strategy based on the use of a central composite design (CCD) with multiple responses. The statistical software Statgraphics Centurion 18-X64 was applied for the generation of the experiment design and data treatment. The data treatment was performed using the analysis of the variance (ANOVA) to check the significance of the studied effects. The concentration of the species used for optimization was as follows: 1 μg L^−1^ V^V^, Hg^II^, Pb^II^ and 2 μg L^−1^ for MetHg, TML.

EDTA, L-cysteine (L-Cys), thiourea, and H_3_PO_4_ were initially selected for the extraction of the analytes from M@GO-TS during the elution step. According to the bibliography, H_3_PO_4_ and EDTA form a complex with vanadium species [21], and L-Cys and thiourea have been previously applied for the elution of Hg, Pb, and their derivatives [16, 27, 31]. Therefore, six online MSPE parameters were identified to be optimized (eluent composition: concentration of thiourea, L-Cys, EDTA, and H_3_PO_4_, and sample and elution flow rates). The first four parameters were optimized using a reduced CCD Draper-and-Lin of 18 experiments [32], which were randomly performed (2^4^ + 2 central points). The selected response functions were the signal-to-noise ratio (s/n) of each species, and the variables to be optimized using the experiments design were those related to the eluent composition (concentration of thiourea, L-Cys, EDTA, and H_3_PO_4_). The concentration ranges studied for each parameter were 1–25 mM, 1–25 mM, 1–12 mM, and 10–60 mM, respectively. Then, sample and elution flow rates were optimized following a univariant strategy. In order to achieve this, the speed of the peristaltic pumps (P1 and P2) and the inner diameter of the pump tubes were modified. The studied ranges were as follows: 1.0–4.8 mL min^−1^ for sample flow rate and between 0.5 and 3.5 mL min^−1^ for eluent flow rate. The desirability function for the multiple response optimization maximizes the response functions (s/n) for the five species. The online MSPE parameters were optimized without HPLC in order to reduce the instrumental complexity of the optimization process. To perform the experiments, FI-ICP MS coupling was used, and each analyte was introduced separately.

According to the bibliography, TBAOH in an acidic medium was selected for mobile phase composition during the separation step. This mobile phase composition has been previously proposed by researchers for the separation of metal ions and their derivatives [16, 21]. Therefore, two parameters were optimized: concentration of TBAOH and pH of phase B (adjusted with H_3_PO_4_ to favor the compatibility between preconcentration and separation systems), in order to maximize the separation of the closest peaks in the chromatogram: the TML and Pb^II^ (R1) and the interference ^35^Cl^16^O^I^ and V^V^ (R2). The studied ranges for each factor were 0.1–0.3 mM and pH 1–6, respectively. The separation parameters were optimized without FI in order to reduce instrumental complexity during the optimization process.

## Results and discussion

### Characterization results

TEM and N_2_ adsorption-desorption isotherm was used to characterize the morphology of the surface. As can be observed in TEM images (Supplementary Fig. SM3), Fe_3_O_4_ nanoparticles were dispersed over the GO sheets with a diameter between 6 and 20 nm. MNPs size was selected intentionally as smaller particles (<6 nm) show reduced saturation magnetization and magnetic susceptibility due to surface effects that low MRI relaxivity values, while larger particles (>20 nm) are difficult to disperse over the GO surface [33]. From the N_2_ adsorption-desorption experiment, a type IV isotherm was obtained, which is typical of mesoporous materials (pore size 20–500 Å). The isotherm of M@GO-TS is shown in Supplementary Fig. SM4, presenting a pore size and specific surface of 83 Å and 48.3 m^2^ g^−1^.

From FT-IR spectra, several characteristic bands can be assigned (Supplementary Fig. SM5). The wide band observed in the 1800–3400 cm^−1^ range was assigned to ν(O-H) from oxygen-containing functional groups of GO. The bands at 1700 cm^−1^ and 1600–1500 cm^−1^ were assigned to ν(C=O) and σ(N-H) from the resulting amide groups of the condensation reaction. The signal at 1200 cm^−1^ was assigned to ν(O-C-O_s_) and ν(O-C-O_as_) derived from the use of sodium chloroacetate during the synthesis process. Moreover, a characteristic band of the selected organic group for the functionalization of the material appeared at 700 cm^−1^, corresponding to ν(C-S). The band at 600 cm^−1^ was assigned to ν(Fe-O), confirming the presence of MNPs on the material surface.

The atomic composition was studied by CHNS elemental analysis and XPS. The results of CNHS analysis are collected in Supplementary Table SM3, and the general XPS spectra are shown in Supplementary Fig. SM6A. As expected, the elements C, O, H, Fe, and S were detected. The presence of Fe and the high concentration of C confirmed the coupling of MNPs with GO, and the S results demonstrated that the material was adequately functionalized. As can be seen in the deconvoluted XPS spectra of the S region (Supplementary Fig. SM6B), the peaks registered at 163.8 eV and 166.1 eV were assigned to Ph-S-S-Ph (79% of total S area) and Ph-SO_2_ (12% of total S area), respectively. This demonstrated that the initial chemical form of the functionalization was modified due to the facility of this organic group to be oxidized in the presence of the GO oxygen-containing functional groups (Supplementary Fig. SM2). This effect was previously described by Chen et al. to prepare reduced GO from GO using L-Cys ((C_3_O_2_NH_6_)-SH) as reductant, obtaining the disulfide derivative cystine (C_3_O_2_NH_6_)-S-S-(C_3_O_2_NH_6_) [34]. The peak at 168.5 eV was assigned to Na_2_SO_4_ (9% of total S area), being justified by the use of H_2_SO_4_ in the synthesis route of GO.

In order to evaluate the magnetic properties of the material, the magnetic susceptibility of both MNPs and M@GO-TS were measured and compared. As can be observed in the VSM curves shown in Supplementary Fig. SM7, the magnetic moment per mg of material for M@GO-TS (2.6 emu g^−1^) was considerably lower than MNPs (6 emu g^−1^). Therefore, it can be concluded that the magnetic properties of the material were compromised due to the coupling of MNPs and non-magnetic material, GO. Despite this inconvenience, the magnetic response of the material M@GO-TS was strong enough to allow the fabrication of the MKR.

### Adsorption capacities of M@GO-TS and alternative materials

As can be seen in Supplementary Table SM1, the calculated adsorption capacities for the majority of the species were considerably high (>5 mg g^−1^ material). This is the case for ^V^V, ^III^Cr, ^II^Mn, ^II^Co, ^II^Cu, ^II^Cd, ^II^Hg, ^II^Pb, MetHg, and TML. Considering the toxicity level of the species and the application of the method for the analysis of environmental and biological samples, ^V^V, ^II^Hg, MetHg, ^II^Pb, and TML were finally selected to develop the analytical method. Regarding the obtained results using M@GO-PSTH and M@GO-DPTH toward ^II^Hg and ^II^Pb (Supplementary Table SM2), M@GO-TS showed better adsorption capacities under the same experimental conditions (10 min of contact at pH 5), especially for ^II^Pb. For this reason, M@GO-TS was considered the best alternative to develop the proposed method.

### Optimization results

The pH curves for the analytes are shown in Supplementary Fig. SM8. The pH dependence of the adsorption on the surface material was demonstrated, being favored at acid pH. However, due to the characteristic chemical properties of the selected species, the pH curves presented different profiles and maximum values. For this reason, pH 3.5 was finally selected as a compromise situation.

**Fig. 3:**
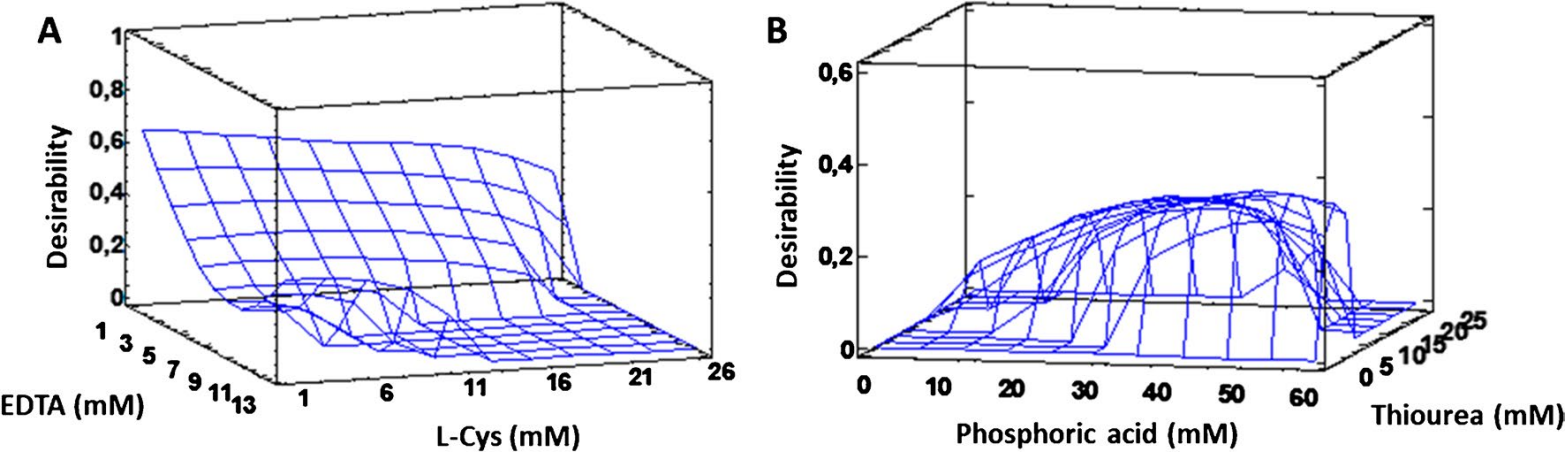
Response surfaces obtained for the optimization of eluent composition. Desirability function maximizes the response function (s/n) for all species: (**A**) desirability in front of EDTA and L-Cys concentration and (**B**) desirability in front of phosphoric acid and thiourea concentrations, respectively

The ANOVA analysis was performed to evaluate the significance of the variables on the response function at the 95% confidence level (*p* < 0.05). The concentration of phosphoric acid was considered the most influential parameter to increase the response function, being statistically significant for V^V^ and Pb^II^. The presence of EDTA negatively affected the signal of TML, and the rest of the parameters were not considered statistically relevant parameters at the confidence level selected. Besides, the resulting surface response can be observed in Fig. 3, which was obtained by attending to the maximum desirability for the response functions. The optimal conditions for eluent composition were as follows: 1 mM L-Cys + 1 mM EDTA + 7 mM thiourea + 40 mM H_3_PO_4_. It can be noted that the concentration values of L-Cys and EDTA proposed by the CCD were the minimum possible (1 mM L-Cys and 1 mM EDTA). For this reason, an extra experiment was performed to compare the results with and without L-Cys and EDTA as eluent components. Better results were obtained when L-Cys and EDTA were not used for the preparation of the eluent (7 mM thiourea + 40 mM H_3_PO_4_), so both reagents were definitely eliminated.

As has been said before, an univariant strategy was followed for the optimization of sample and eluent flow rates of the online MSPE process, and TBAOH concentration and pH for the preparation of the mobile phase. As a result, 2.7 ml min^−1^ and 1.5 ml min^−1^ were selected as the optimum flow rates values (Supplementary Figs. SM9 and SM10), respectively, and 0.15 mM TBAOH in H_3_PO_4_ pH 4.5 was the final composition of mobile phase B (Supplementary Figs. SM11 and SM12). In general, the signals were considerably lower when the sample flow rate increased for all species (Supplementary Fig. SM9). This effect can be explained by attending to the kinetics of the adsorption process, which was considered too slow to allow efficient retention of the analytes when the sample passed through the MKR at high flow rates. In order to establish a compromise situation between the sample volume per minute that can be introduced in the online MSPE system and the signal loss, a medium sample flow rate value was selected (2.7 mL min^−1^). Besides, the results shown in Supplementary Fig. SM11 indicated that TBAOH concentration was not especially relevant for the separation of the species. The optimization results of all parameters are summarized in Table 2.

**Table 2:** Optimization results obtained from the multivariant and univariant optimization strategies

Parameter	Range	Units	Strategy	Optimum value
Sample pH	1–12	-	Univariant	3.5
Phosphoric acid	10–60	mM	Multivariant	40
Thiourea	1–12	mM	Multivariant	7
L-Cys	1–25	mM	Multivariant	-
EDTA	1–25	Mm	Multivariant	-
Sample flow rate	1.0–4.8	mL min^−1^	Univariant	2.7
Elution flow rate	0.5–3.5	mL min^−1^	Univariant	1.5
Mobile phase pH	1–6	-	Univariant	4
TBAOH	0.1–0.3	Mm	Univariant	0.16

Under the optimum conditions, the sample loading time was studied with a standard solution of 0.5 μg L^−1^ between 5 and 20 min for each specie. The signal intensities were increased practically linearly in all the time intervals. A sample loading time of 10 min was chosen in order to obtain good enrichment factors in a short time. If necessary, a longer sample loading time can be used.

### Reuse of the reactor

The MKR was subjected to successive adsorption/elution cycles in order to evaluate the reusability of the adsorbent material. The preconcentration processes and measurements were performed as explained above using a mix of 1 μg L^−1^ at pH 3.5 of the selected species. The results were monitored until an obvious signal loss was observed. The MKR showed high long-term stability and reusability, being reused in more than 200 adsorption-elution cycles.

### Analytical performance

The analytical performance of the method was studied under optimum conditions, and the results are summarized in Table 3. As can be observed, the method presented a wide linear range for all analytes. The calibration curves were defined as *y* = *bx* + *a*, and the LODs and LOQs were calculated as 3.3S_a_/b and 10S_a_/b, respectively. The parameter *b* was the slope of the calibration curve, while *S*_*a*_ was identified as the intercept uncertainty, being considered the standard deviation of the blank. In order to calculate the precision of the method, a solution containing 0.5 μg L^−1^ of all species was measured six times to calculate %RSD. The enrichment factor (EF) was estimated as the ratio of the sample volume introduced (27 mL) and the eluent volume collected in the chromatographic vial (1 mL). Then, the EF value of the method was calculated as 27, which is suitable for an online MSPE system. Better analytical performances (LODs, LOQs, and EF) can be obtained, if necessary, by simply increasing the sample loading time.

**Table 3:** Analytical performance of the method

Parameters	Analytical Features				
	Pb^II^	TML	Hg^II^	MetHg	V^V^
Studied linear range (μg L^−1^)	0.02-2	0.05-2	0.005-2	0.05-2	0.005-2
Slope	2.04 × 10^7^	1.54 ×10^5^	2.66 × 10^5^	1.34 × 10^5^	1.79 × 10^7^
Interception	218762	523071	72.333	12080	1009
Intercept uncertainty, Sa	3.20 × 10^4^	788	162	458	2.26 × 10^3^
LOD (μg L^−1^)	0.005	0.02	0.002	0.01	0.0004
LOQ (μg L^−1^)	0.016	0.05	0.006	0.03	0.001
Repeatability, RSD (%)	2.8	3.0	4.5	2.5	4.0
Enrichment factor	27	27	27	27	27

A comparative study was performed in order to highlight the main advantages and disadvantages of the proposed method (Table 4). In our work, the MSPE was performed in an online mode, and five organic and inorganic species of three elements, Hg, Pb, and V, were determined at the same time. Compared with the in-batch methodologies, this methodology provides better enrichment factors and, so, improved LOD and LOQ because greater sample volumes and contact times between phases are used. In the work of Li et al., an in-batch MSPE procedure was applied, and two species of Hg were determined with better LODs and LOQs. In that work, 400 mL of sample was used, and the contact time between the phases was 30 min for loading time and 10 min for elution time [37]. However, this type of methodology cannot be automatized.

**Table 4:** Comparative study of chromatographic speciation methods found in the bibliography

Technique material	Application	Sample (mL)	LOD (μg L^−1^)					LOQ (μg L^−1^)					Ref.
			Pb^II^	TML	Hg^II^	MetHg	V^V^	Pb^II^	TML	Hg^II^	MetHg	V^V^	
HPLC-ICP MS -	Particulate matter	-	-	-	-	-	0.06	-	-	-	-	Not reported	[35]
HPLC-UV vis -	Water	-	0.075	-	0.120	-	-	0.225	-	0.450	-	-	[36]
HPLC-ICP MS -	Lotus seeds	-	0.10	0.0076	0.023	0.025	-	1.34*	0.11*	0.31*	0.31*	-	[17]
Offline MSPE/HPLC-ICP MS Dithizone functionalized MGO	Environmental water and fish samples	400			0.00048	0.00017				0.0016	0.00057		[37]
Online SPE/HPLC-ICP MS GO@SiO_2_	River and tap water	10	-	0.000018	-	-	-	-	0.000058	-	-	-	[38]
MSPE/GC-AFS** Cellulose MNPs	River and tap water	15	-	-	0.0056	0.004	-	-	-	0.186	0.133	-	[39]
Online MSPE/HPLC-ICP MS M@GO-TS	Urine and seawater	27	0.005	0.02	0.002	0.01	0.0004	0.016	0.05	0.006	0.03	0.001	This work

*μg Kg^−1^

**GC-AFS: gas chromatography coupled with atomic fluorescence detection

On the other hand, our method presented lower LODs than other chromatographic methods with no preconcentration process prior to detection (including methods with ICP MS as a detector) [17, 35, 36]. Therefore, it can be concluded that the dilution due to the mobile phase, generated when HPLC is coupled, has been adequately compensated with a simple and automatic MSPE. From the comparative study, it can be deduced that better performance can be obtained for inorganic species with functionalized MGO. For example, Yang et al. considered the interaction of GO@SiO_2_ with Pb^II^ too weak to develop an online SPE [38], and only organic species of lead were speciated (TEL and TML). This fact was in good agreement with the results shown in the work developed by Abujaber et al. [39], where the LOD for MetHg was lower, while the LOD for Hg^II^ is higher than those obtained with our method. The fact of the magnetic graphene oxide (M@GO), both MNPs and GO have been functionalized in our material, allows both types of interactions: physical (as in the work of Yang et al. or Abujaber et al.) and chemical by means of a chemical bond between the functional group and the inorganic ions. For this reason, M@GO-TS adsorbs both inorganic and organic species. The method presented in this paper has also shown a high versatility to be applied for the analysis of complex matrix samples such as seawater and urine with external calibration. Furthermore, the proposed analytical method allowed the simultaneous preconcentration of metallic and organometallic species of Pb, Hg, and V using an automatized MSPE process prior to analysis by HPLC-ICP MS, being the first of these characteristics in the knowledge area.

### Validation

The optimized method was applied to the analysis of real samples (urine and Malaga seawater) and certified samples (Fortified Lake Water TMDA 64.3) for validation purposes. The accuracy of the method was studied by calculating the recovery of the spike test in real samples and by comparing the total Pb found in the TMDA 64.3 with the certified value. There are few certified samples useful for the validation of speciation procedures, especially in the cases of species of Hg and V. Recovery analysis is an accepted alternative to demonstrate the accuracy of a method when there are no other alternatives, as has been in this case. As can be observed in Table 5, the spike tests were satisfactory, obtaining high recoveries values close to 100% for all samples. As expected, both metal ions and organometallic species were determined in the environmental sample, while metal ions were not found in urine samples. The total Pb concentration found in the TMDA 64.3 was calculated as 297 ± 4 μg L^−1^ (297 ± 4 μg L^−1^ of Pb^II^ and <LOD for TML), which was in good agreement with the certified value (279 ± 22 μg L^−1^). The estimated standard deviations for recovery tests and the determined certified value were generally in agreement with the %RSD of the method (2.5–4.0%). Moreover, no significant differences between the certified and found value were observed by t statistical test. Both certified and real samples presented complex matrices with high content of potential interferences such as other transition metals and salinity. Therefore, it can be concluded that the proposed method did not suffer from interferences of this kind of samples, which were analyzed using external calibration. The polyatomic interference of ^35^Cl^16^O^I^ for ^51^V is well known. However, our method separated both species, and no peak overlap was detected. This characteristic was especially relevant during the analysis of Málaga seawater. Despite the high complexity of the matrices, the interferences were avoided due to the combination of a selective extraction process and an optimized chromatographic program.

**Table 5:** Real samples analysis (*n* = 3)

Sample	Added (μg L^−1^)					Found (μg L^−1^)					Recovery (%)				
	Pb^II^	TML	Hg^II^	MetHg	V^V^	Pb^II^	TML	Hg^II^	MetHg	V^V^	Pb^II^	TML	Hg^II^	MetHg	V^V^
Urine 1	-	-	-	-	-	<LOD	<LOD	<LOD	<LOD	<LOD	-	-	-	-	-
	0.3	0.3	0.3	0.3	0.3	0.27 ± 0.02	0.315 ± 0.013	0.27 ± 0.08	0.2922 ± 0.0014	0.32 ± 0.11	90	105	90	97	106
	0.5	0.5	0.5	0.5	0.5	0.52 ± 0.10	0.486 ± 0.009	0.51 ± 0.03	0.467 ± 0.007	0.53 ± 0.02	104	97	102	93	106
Urine 2	-	-	-	-	-	<LOD	0.042 ± 0.011	<LOD	0.0572 ± 0.0009	<LOD	-	-	-	-	-
	0.3	0.3	0.3	0.3	0.3	0.27 ± 0.04	0.29 ± 0.03	0.28 ± 0.02	0.30 ± 0.04	0.29 ± 0.02	90	97	93	100	97
	0.5	0.5	0.5	0.5	0.5	0.56 ± 0.02	0.538 ± 0.019	0.49 ± 0.04	0.43 ± 0.08	0.47 ± 0.02	112	108	98	86	94
Seawater	-	-	-	-	-	<LOD	0.263 ± 0.003	<LOD	1.203 ± 0.011	0.10 ± 0.02	-	-	-	-	-
	0.3	0.3	0.3	0.3	0.3	0.27 ± 0.07	0.51 ± 0.04	0.36 ± 0.03	1.50 ± 0.02	0.38 ± 0.03	90	83	83	100	93
	0.5	0.5	0.5	0.5	0.5	0.45 + 0.02	0.84 ± 0.02	0.47 ± 0.07	1.71 ± 0.02	0.62 ± 0.03	90	114	94	102	102

## Conclusions

A novel nanomaterial based on a double functionalization of a patented MGO by the research group named M@GO was developed and characterized (M@GO-TS). This material was applied for the optimization and validation of an online MSPE process prior to HPLC-ICP MS analysis. The greater functionalization of M@GO-TS has provided good adsorption of both inorganic and organometallic species. An MSPE method has been proposed for the simultaneous and automatic preconcentration of five metallic species of highly toxic elements and their elution with only 1 mL of the eluent. The experimental conditions for the HPLC-ICP MS determinations were also optimized. The new method has been proven to be promising for the routine monitoring of inorganic and organic species of V, Hg, and Pb in environmental waters and biological samples such as seawater and human urine. Collision or reaction cells, washing steps, or salinity adjustment were not necessary. This method was considered free of interferences thanks to a preconcentration/separation pretreatment combined with an adequate chromatographic system, allowing the separation of the polyatomic interference ^35^Cl^16^O^I^ of V^V^ and the use of external calibration. A total of nine parameters related to the online MSPE process and the HPLC-ICP MS system were optimized in order to maximize the signals and the resolution of the closest chromatographic peaks. The analytical method presented good sensitivity and precision, with the following LODs and RSDs for the species Pb^II^, TML, Hg^II^, MetHg, and V^V^: 0.005 μg L^−1^, 0.02 μg L^−1^, 0.002 μg L^−1^, 0.09 μg L^−1^, and 0.0004 μg L^−1^ and 2.8%, 3%, 4.5%, 2.5%, and 4%, respectively. The performance of the optimized method has been compared with other HPLC-ICP MS methods described in the bibliography for the speciation and determination of Hg, Pb, or V. Comparing with the in-batch methodologies, these provide better enrichment factors, and so, improved LOD and LOQ, because greater sample volumes and contact times between phases are used, but this type of methodology cannot be automatized and higher analysis time is required. Obviously, the sensibility of this method is better than those found for other HPLC-ICP MS without preconcentration. The fact that both MNPs and GO have been functionalized in our material (M@GO-TS) allows two types of interactions: physical and chemical, by means of a chemical bond between the functional group and the inorganic ions. For this reason, M@GO-TS adsorbs both inorganic and organic species, showing better sensitivity toward inorganic species than other online MSPE-HPLC-ICP MS. A reference material (fortified lake water TMDA 64.3) and three real samples (seawater and two human urine samples) were used for the validation of the method, obtaining results according to the certified value and recoveries close to 100%. The MKR fabricated with the nanomaterial M@GO-TS demonstrated good stability and resistance, being refilled only three times during the development of this work. To the best of our knowledge, this is the first method based on an automatic MSPE process combined with HPLC-ICP MS for multielemental determination of organic and inorganic species of mercury, lead, and V. Moreover, it has been demonstrated that the initial TS structure has been modified due to the presence of GO in the material, and this is the first report of this effect in the TS group, being also applied as a functional group attached to the surface of the sorbent. The analytes (organic and inorganic species of highly toxic elements) could be simultaneously collected and preconcentrated in situ with the automatic MSPE procedure. Thanks to the stability of the eluate containing the target analytes collected in the vial (at least 24 h), this solution could be carried to the laboratory for its injection in the optimized multielemental HPLC-ICP MS method. The proposed method provides an automatic monitoring system to control highly toxic species in seas and oceans with continuous and automatic sampling and pretreatment of the samples prior to the speciation analysis in the laboratory.

## Supplementary information

ESM 1 (DOCX 2486 kb)

## References

[1] Chen G, Lai B, Mei N, Liu J, Mao X (2017). Mercury speciation by differential photochemical vapor generation at UV-B vs. UV-C wavelength. Spectrochim Acta Part B At Spectrosc.

[2] Kim KH, Kabir E, Jahan SA (2016). A review on the distribution of Hg in the environment and its human health impacts. J Hazard Mater.

[3] Yuan X, Yang G, Ding Y, Li X, Zhan X, Zhao Z, Duan Y (2014). An effective analytical system based on a pulsed direct current microplasma source for ultra-trace mercury determination using gold amalgamation cold vapor atomic emission spectrometry. Spectrochim Acta Part B At Spectrosc.

[4] Leopold K, Foulkes M, Worsfold P (2010). Methods for the determination and speciation of mercury in natural waters—a review. Anal Chim Acta.

[5] Zhu S, Chen B, He M, Huang T, Hu B (2017). Speciation of mercury in water and fish samples by HPLC-ICP-MS after magnetic solid phase extraction. Talanta.

[6] Gidlow DA (2004). Lead toxicity. Occup Med (Chic Ill).

[7] Comitre ALD, Reis BF (2005). Automatic flow procedure based on multicommutation exploiting liquid-liquid extraction for spectrophotometric lead determination in plant material. Talanta.

[8] Chauhan AS, Bhadauria R, Singh AK, Lodhi SS, Chaturvedi DK, Tomar VS (2010). Determination of lead and cadmium in cosmetic products. J Chem Pharm Res.

[9] Chen J, Xiao S, Wu X, Fang K, Liu W (2005). Determination of lead in water samples by graphite furnace atomic absorption spectrometry after cloud point extraction. Talanta.

[10] Saçmacı Ş, Saçmacı M (2020) The rapid determination of lead in food samples by magnetic dispersive solid-phase extraction coupled zeta potential analyzer. Int J Environ Anal Chem. 10.1080/03067319.2020.1784885

[11] Leal-Granadillo IA, Alonso JIG, Sanz-Medel A (2000). Determination of the speciation of organolead compounds in airborne particulate matter by gas chromatography–inductively coupled plasma mass spectrometry. Anal Chim Acta.

[12] World Health Organization (2011) Lead in drinking-water. WHO/SDE/WSH/03.04/09/Rev/1, Geneva

[13] Chen ZL, Owens G (2008). Trends in speciation analysis of vanadium in environmental samples and biological fluids—a review. Anal Chim Acta.

[14] Thompson KH, Orvig C (2006). Vanadium in diabetes: 100 years from Phase 0 to Phase I. J Inorg Biochem.

[15] Ghorbani-Kalhor E, Hosseinzadeh-Khanmiri R, Abolhasani J, Babazadeh M, Hassanpour A (2015). Determination of mercury(II) ions in seafood samples after extraction and preconcentration by a novel functionalized magnetic metal-organic framework nanocomposite. J Sep Sci.

[16] Song Y, Ma Q, Cheng H, Liu J, Wang Y (2021). Simultaneous enrichment of inorganic and organic species of lead and mercury in pg L−1 levels by solid phase extraction online combined with high performance liquid chromatography and inductively coupled plasma mass spectrometry. Anal Chim Acta.

[17] Zhang D, Yang S, Ma Q, Sun J, Cheng H, Wang Y, Liu J (2020). Simultaneous multi-elemental speciation of As, Hg and Pb by inductively coupled plasma mass spectrometry interfaced with high-performance liquid chromatography. Food Chem.

[18] Cheng H, Zhang W, Wang Y, Liu J (2018). Graphene oxide as a stationary phase for speciation of inorganic and organic species of mercury, arsenic and selenium using HPLC with ICP-MS detection. Microchimica Acta.

[19] Caumette G, Lienemann C-P, Merdrignac I, Bouyssiere B, Lobinski R (2010). Fractionation and speciation of nickel and vanadium in crude oils by size exclusion chromatography-ICP MS and normal phase HPLC-ICP MS. J Anal At Spectrom.

[20] Ščančar J, Berlinger B, Thomassen Y, Milačič R (2015). Simultaneous speciation analysis of chromate, molybdate, tungstate and vanadate in welding fume alkaline extracts by HPLC–ICP-MS. Talanta.

[21] Kilibarda N, Afton SE, Harrington JM, Yan F, Levine KE (2013). Rapid speciation and determination of vanadium compounds using ion-pair reversed-phase ultra-high-performance liquid chromatography inductively coupled plasma-sector field mass spectrometry. J Chromatogr A.

[22] Montoro-Leal P, García-Mesa JC, Morales-Benítez I, García de Torres A, Vereda Alonso E (2021). Semiautomatic method for the ultra-trace arsenic speciation in environmental and biological samples via magnetic solid phase extraction prior to HPLC-ICP-MS determination. Talanta.

[23] Morales-Benítez I, Montoro-Leal P, García-Mesa JC, Verdeja-Galán J, Vereda Alonso EI (2022). Magnetic graphene oxide as a valuable material for the speciation of trace elements. TrAC Trends Anal Chem.

[24] Turiel E, Díaz-Álvarez M, Martín-Esteban A (2020). Surface modified-magnetic nanoparticles by molecular imprinting for the dispersive solid-phase extraction of triazines from environmental waters. J Sep Sci.

[25] Haniffa MACM, Ching YC, Illias HA, Munawar K, Ibrahim S, Nguyen DH, Chuah CH (2021). Cellulose supported promising magnetic sorbents for magnetic solid-phase extraction: a review. Carbohydr Polym.

[26] Montoro Leal P, García Mesa JC, López Guerrero MM, Vereda Alonso EI (2021) Metal-adsorbing composite material based on magnetic graphene oxide and method for obtaining same. Oficina Española de Patentes y Marcas n° pub.: 2 844 942. European patent application EP 4 095 097 A1. https://patentimages.storage.googleapis.com/67/1e/17/376ef60a39c6ca/ES2844942B2.pdf. Accessed 24 Nov 2021

[27] García-Mesa JC, Montoro Leal P, López Guerrero MM, Vereda Alonso EI (2019). Simultaneous determination of noble metals, Sb and Hg by magnetic solid phase extraction on line ICP OES based on a new functionalized magnetic graphene oxide. Microchem J.

[28] Diagboya PN, Olu-Owolabi BI, Zhou D, Han B-H (2014). Graphene oxide–tripolyphosphate hybrid used as a potent sorbent for cationic dyes. Carbon N Y.

[29] González Moreno A, López Guerrero MM, Vereda Alonso E, García de Torres A, Cano Pavón JM (2017). Development of a new FT-IR method for the determination of iron oxide. Optimization of the synthesis of suitable magnetic nanoparticles as sorbent in magnetic solid phase extraction. New J Chem.

[30] Montoro-Leal P, García-Mesa JC, Lopez Guerrero MDM, Vereda Alonso E (2020). Comparative study of synthesis methods to prepare new functionalized adsorbent materials based on MNPs–GO coupling. Nanomaterials.

[31] Mashhadizadeh MH, Amoli-Diva M, Shapouri MR, Afruzi H (2014). Solid phase extraction of trace amounts of silver, cadmium, copper, mercury, and lead in various food samples based on ethylene glycol bis-mercaptoacetate modified 3-(trimethoxysilyl)-1-propanethiol coated Fe3O4 nanoparticles. Food Chem.

[32] Gámiz-Gracia L, Cuadros-Rodríguez L, Almansa-López E, Soto-Chinchilla JJ, García-Campaña AM (2003). Use of highly efficient Draper-Lin small composite designs in the formal optimisation of both operational and chemical crucial variables affecting a FIA-chemiluminescence detection system. Talanta.

[33] Ruiz A, Salas G, Calero M, Hernández Y, Villanueva A, Herranz F, Veintemillas-Verdaguer S, Martínez E, Barber DF, Morales MP (2013). Short-chain PEG molecules strongly bound to magnetic nanoparticle for MRI long circulating agents. Acta Biomater.

[34] Chen D, Li L, Guo L (2011). An environment-friendly preparation of reduced graphene oxide nanosheets via amino acid. Nanotechnology.

[35] Taira M, Sakakibara K, Saeki K, Ohira SI, Toda K (2020). Determination of oxoanions and water-soluble species of arsenic, selenium, antimony, vanadium, and chromium eluted in water from airborne fine particles (PM 2.5): effect of acid and transition metal content of particles on heavy metal elution. Environ Sci Process Impacts.

[36] Thirumalai M, Kumar SN, Prabhakaran D, Sivaraman N, Maheswari MA (2018). Dynamically modified C18 silica monolithic column for the rapid determinations of lead, cadmium and mercury ions by reversed-phase high-performance liquid chromatography. J Chromatogr A.

[37] Li L, Bi R, Wang Z, Xu C, Li B, Luan L, Chen X, Xue F, Zhang S, Zhao N (2019). Speciation of mercury using high-performance liquid chromatography-inductively coupled plasma mass spectrometry following enrichment by dithizone functionalized magnetite-reduced graphene oxide. Spectrochim Acta Part B At Spectrosc.

[38] Yang S, Song Y, Ma Q, Cheng H, Wang Y, Liu J (2020). Quantification of ultra-trace organolead species in environmental water by inductively coupled plasma mass spectrometry with online solid-phase extraction and high performance liquid chromatographic separation. Anal Chim Acta.

[39] Abujaber F, Jiménez-Moreno M, Guzmán Bernardo FJ, Rodríguez Martín-Doimeadios RC (2019). Simultaneous extraction and preconcentration of monomethylmercury and inorganic mercury using magnetic cellulose nanoparticles. Microchimica Acta.

