# Supplementary material for: Synthesis of a novel magnetic nanomaterial for the development of a multielemental speciation method of lead, mercury, and vanadium via HPLC-ICP MS
Source: Mikrochim Acta. 2023 Jul 17;190(8):296. doi: 10.1007/s00604-023-05877-x (PMC10352391; doi:10.1007/s00604-023-05877-x)
Supplement: Supplementary file 1 — (DOCX 2486 kb) [file 604_2023_5877_MOESM1_ESM.docx]

**Supplementary material**

**Synthesis of a novel magnetic nanomaterial for the development of a semiautomatic multielemental speciation method** **of lead, mercury and vanadium via HPLC-ICP MS**

P. Montoro-Leal^1‡^, J.C. García-Mesa^1‡^, I. Morales-Benítez^1‡^, L. Vázquez-Palomo^1^, M.M. López Guerrero^1^, E.I. Vereda Alonso^1*^

^1^ Department of Analytical Chemistry, Faculty of Sciences, University of Malaga, 29071, Málaga, Spain

^‡^ Equally contribution

*Corresponding author: [eivereda@uma.es](mailto:eivereda@uma.es)

**Functionalization of M@GO**

0.35 g of M@GO is suspended in 60 mL of ethanol 0.8% MTS. The mixture was kept at reflux at for 8 h. The resulting product is washed three times with methanol and dried at 50 ⁰C. The structure of functionalized M@GO is showed in Fig. SM1 and SM2. The final functionalization step is due to the facility of the introduced functionalization group to be oxidized, which is discussed in optimization results section.


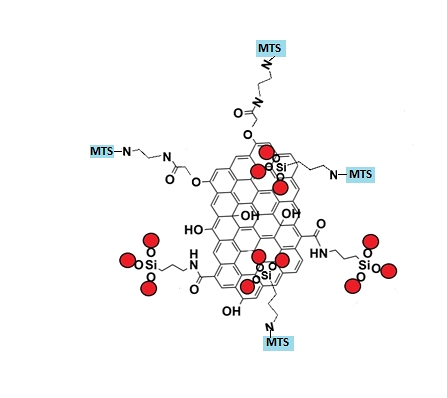


**Figure SM1** Structure of functionalized M@GO


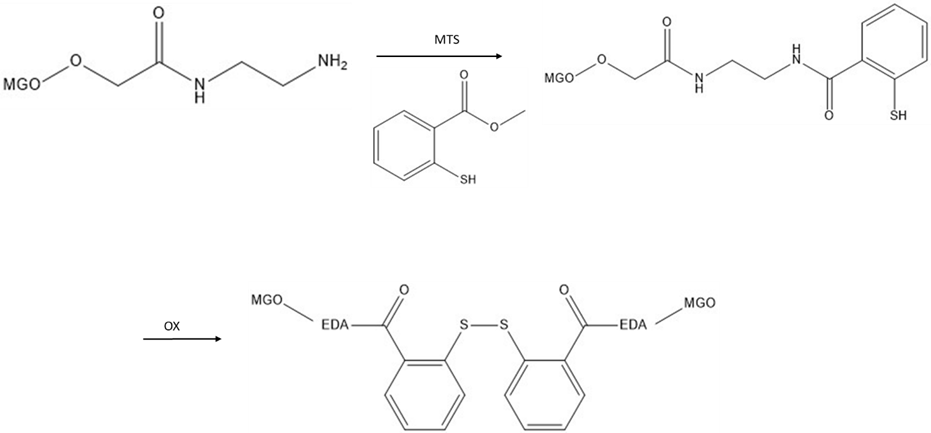


**Figure SM2** Functionalization process M@GO to obtain M@GO-TS

**Adsorption capacities of M@GO-TS**

**Table SM1** Adsorption capacities (mg g^-1^) of M@GO-TS towards metal ions and derivatives at different pHs

| **Species** | **pH 1** | **pH 5** | **pH 8** |
| --- | --- | --- | --- |
| ^V^V | 2.0 | 7.8 | 5.8 |
| ^III^Cr | 1.5 | 5.6 | 10 |
| ^II^Mn | 0.0 | 0.0 | 6.5 |
| ^II^Co | 0.9 | 7.0 | 9.7 |
| ^II^Cu | 1.0 | 7.0 | 9.8 |
| ^V^As | 0.0 | 1.7 | 1.5 |
| ^II^Cd | 0.5 | 2.5 | 9.9 |
| ^V^Sb | 0.0 | 0.6 | 0.0 |
| ^II^Hg | 8.1 | 7.9 | 8.4 |
| ^II^Pb | 0.0 | 6.3 | 0.0 |
| MetHg | 5.0 | 8.0 | 5.1 |
| TML | 6.4 | 4 | 3.2 |

**Table SM2** Adsorption capacities of M@GO-TS, M@GO-DPTH and M@GO-PSTH towards mercury and lead metal ions at pH 5

| **Species** | **M@GO-TS** | **M@GO-DPTH** | **M@GO-PSTH** |
| --- | --- | --- | --- |
| ^II^Hg | 7.9 | 6.3 | 7.5 |
| ^II^Pb | 6.3 | 0.8 | 1.4 |

**
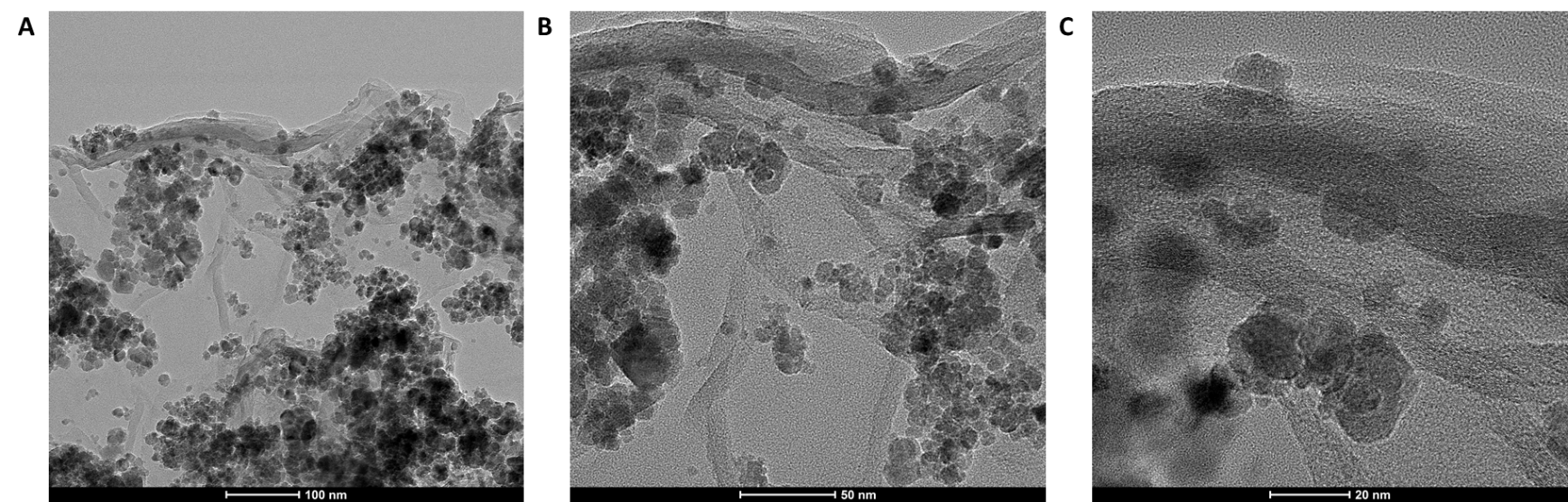
Characterization results**

**Figure SM3** TEM images of M@GO-TS with a scale of A) 100 nm, B) 50 nm and C) 20 nm

**
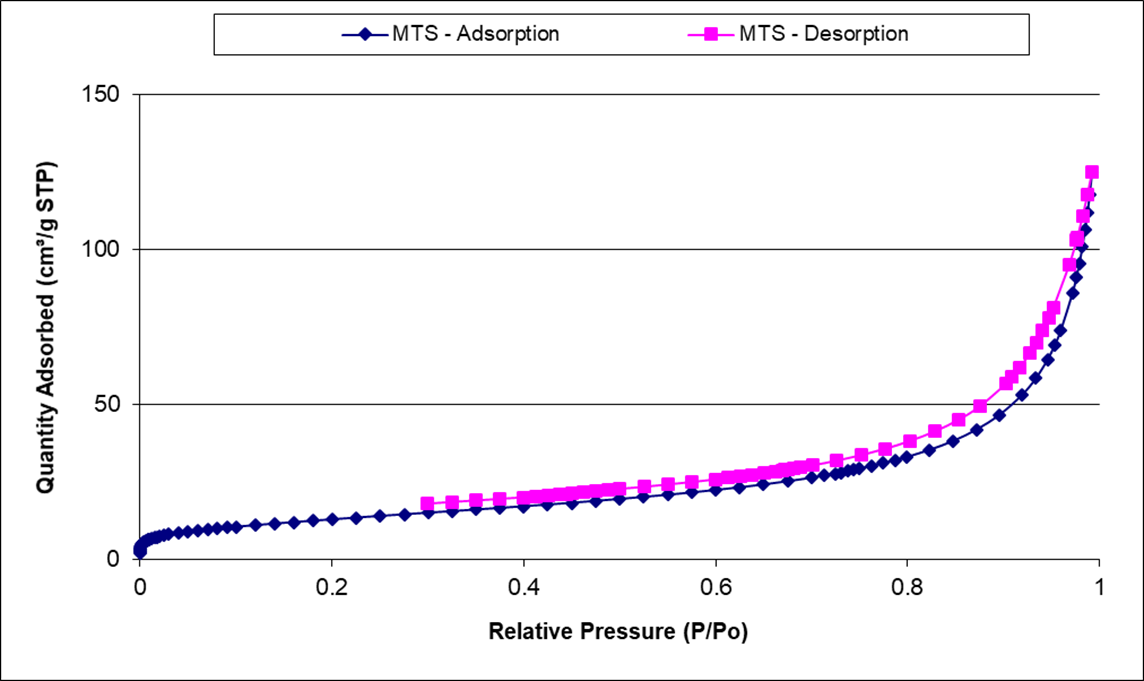
**

**Figure SM4** N_2_ adsorption-desorption isotherm of M@GO-TS

**
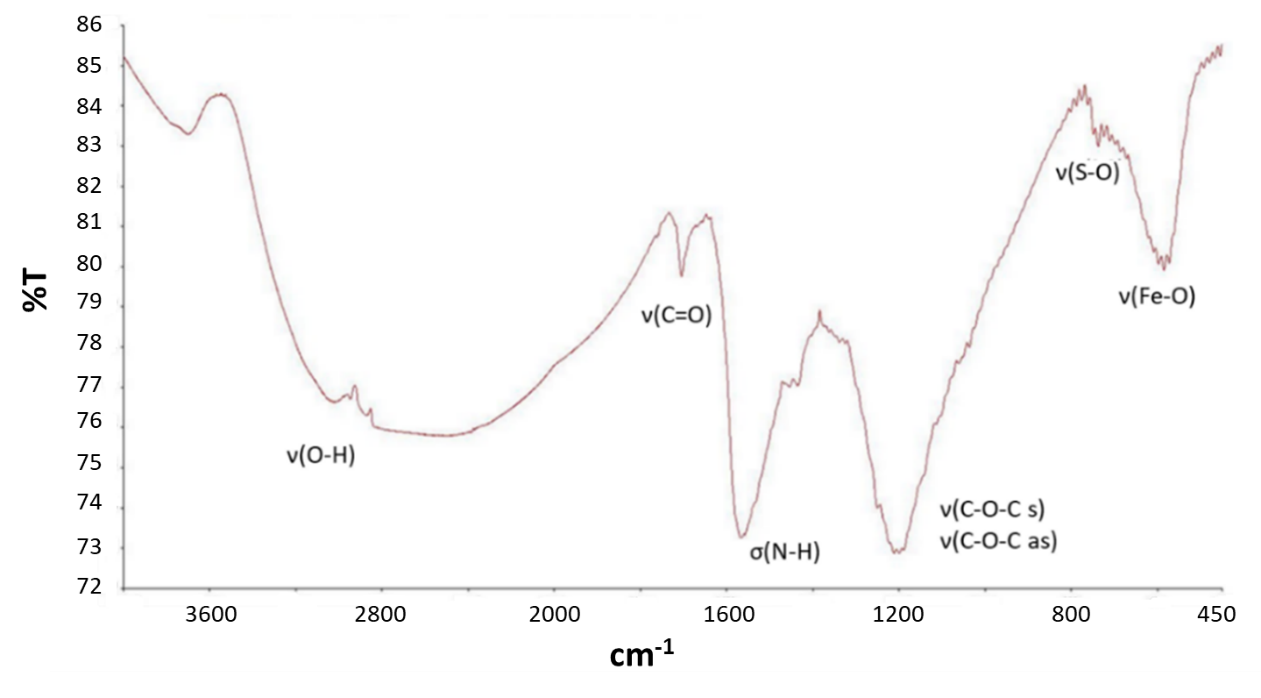
**

**Figure SM5** IR-FT spectra of M@GO-TS

**Table SM3** Atomic composition of M@GO-TS by CHNS elemental analysis

| **% C** | **% H** | **% N** | **% S** |
| --- | --- | --- | --- |
| 35 | 1.9 | 3.8 | 1.5 |

**
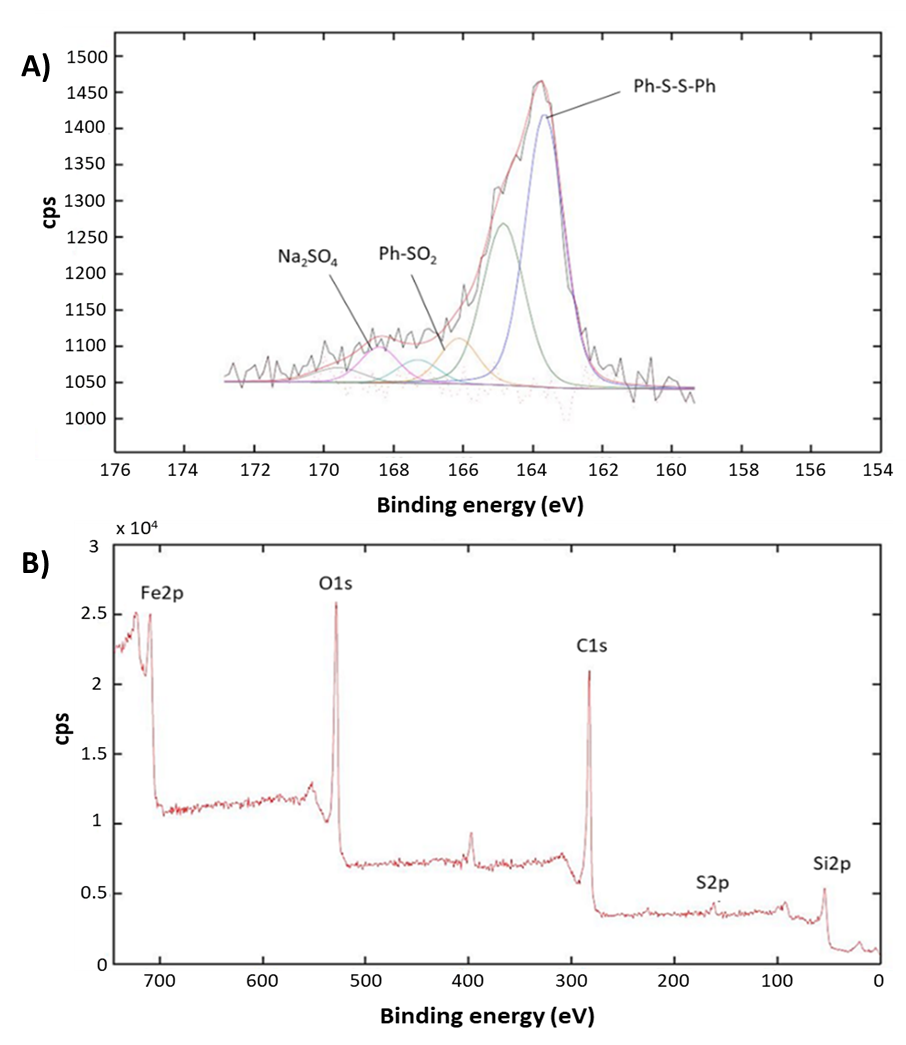
**

**Figure SM6** XPS results A) complete range spectra of M@GO (0-750 eV) and B) spectral region of S.

**
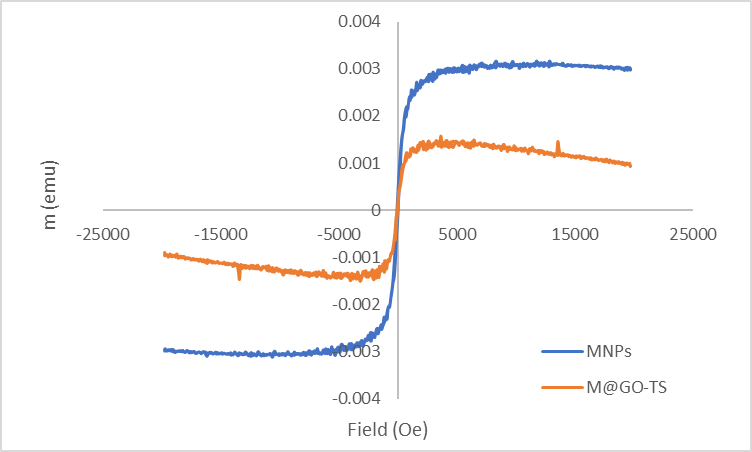
**

**Figure SM7** VSM curves of MNPs and M@GO-TS

**
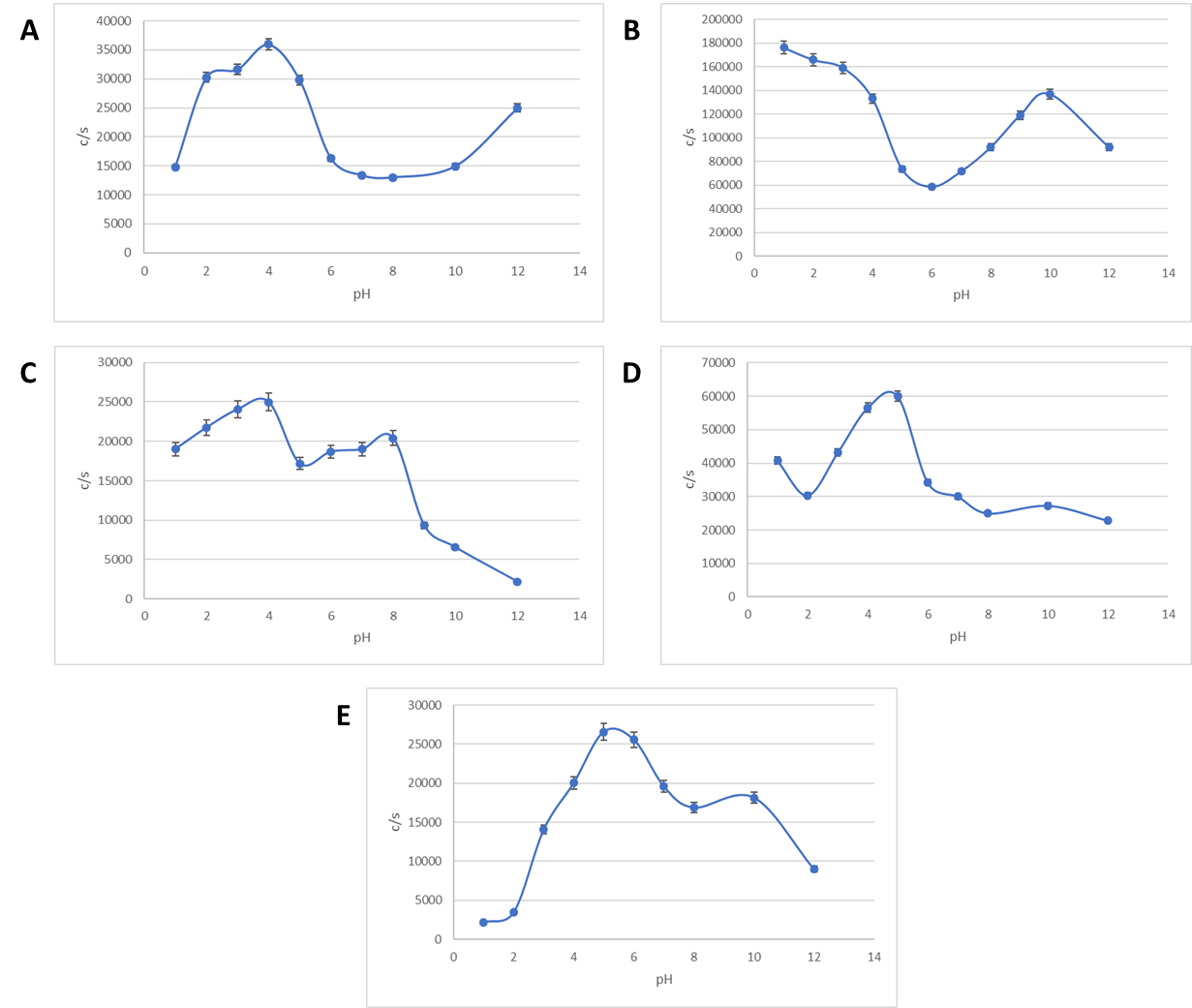
Optimization results**

**Figure SM8** pH curves for A) Pb^II^, B) TML, C) Hg^II^, D) MetHg, E) V^V^


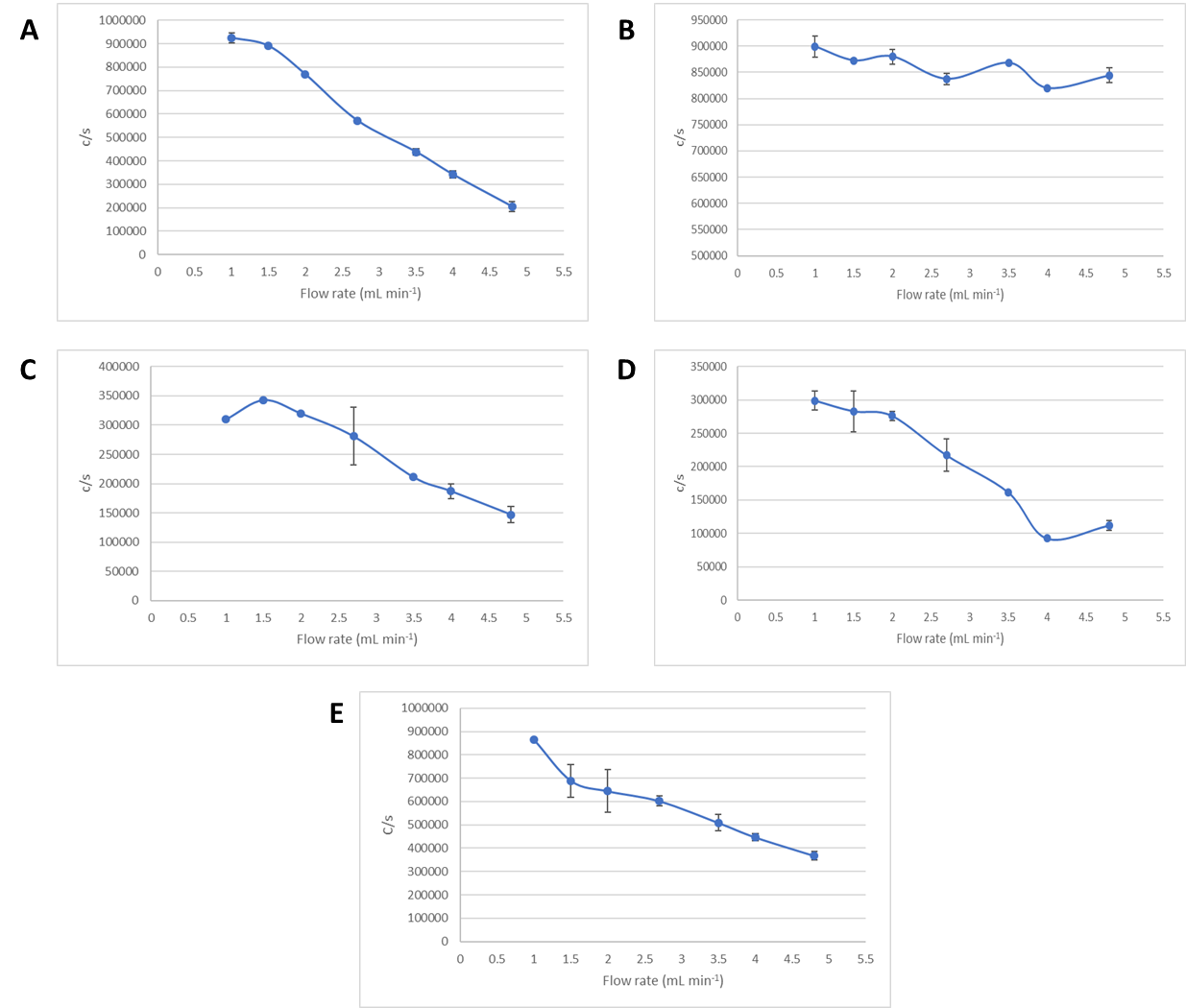


**Figure SM9** Optimization results of sample flow rates for A) Pb^II^, B) TML, C) Hg^II^, D) MetHg, E) V^V^


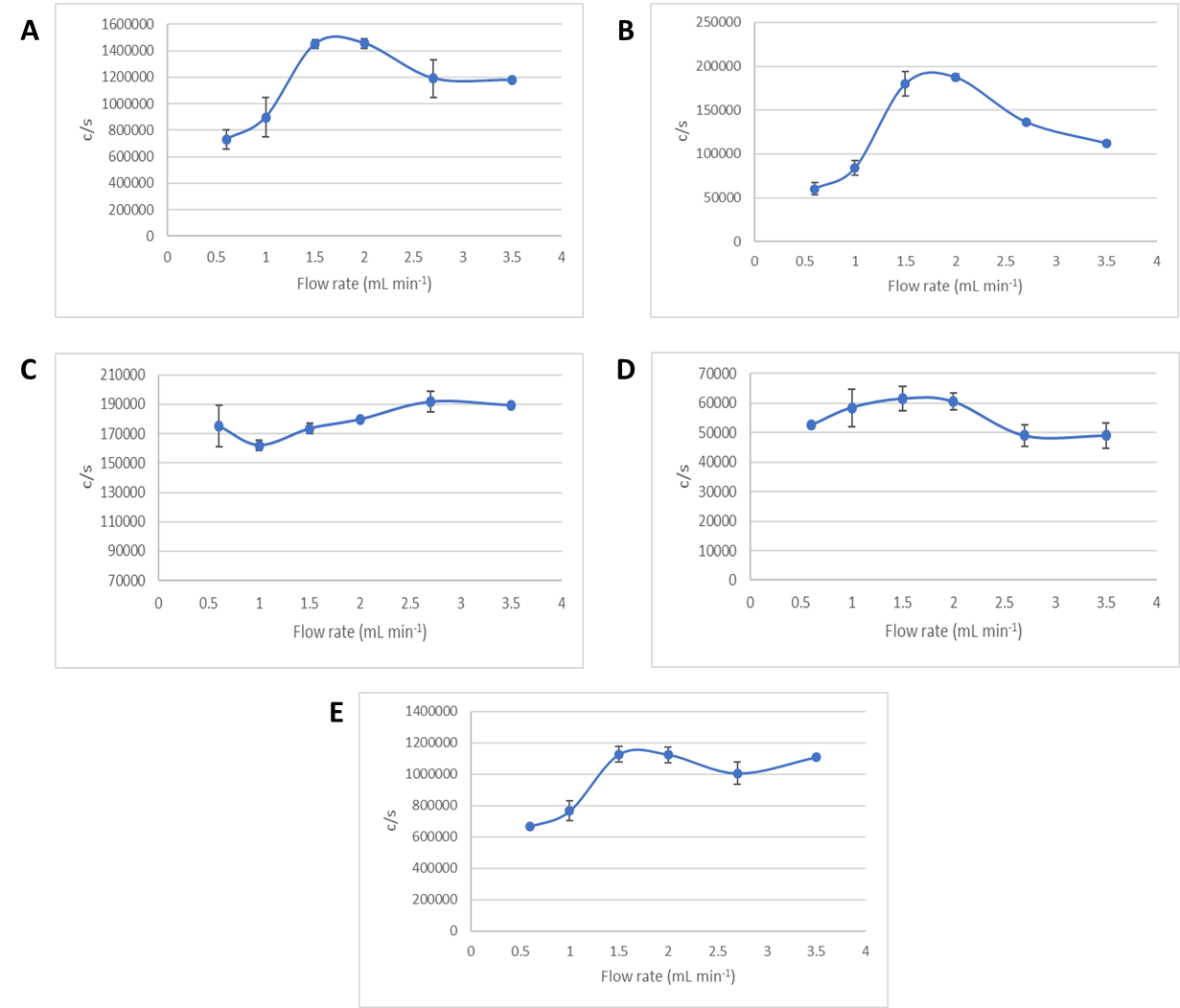


**Figure SM10** Optimization results of elution flow rates for A) Pb^II^, B) TML, C) Hg^II^, D) MetHg, E) V^V^


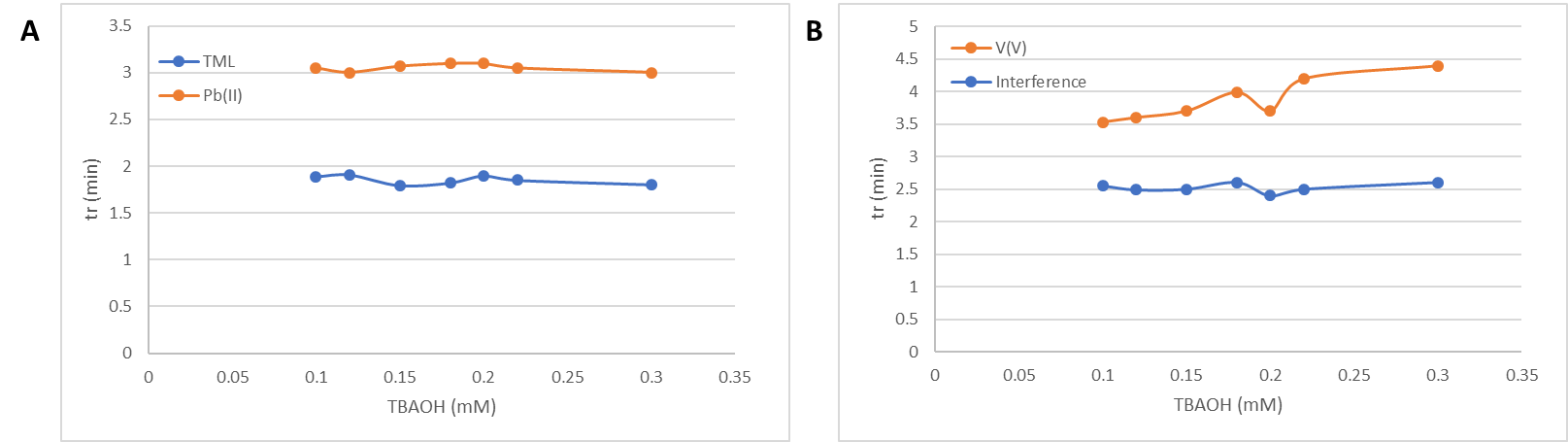


**Figure SM11** Optimization results of TBAOH concentration in order to maximize A) R1 (distance between retention times for TML and Pb(II), and B) R2 (distance between retention times for V(V) and its interference).


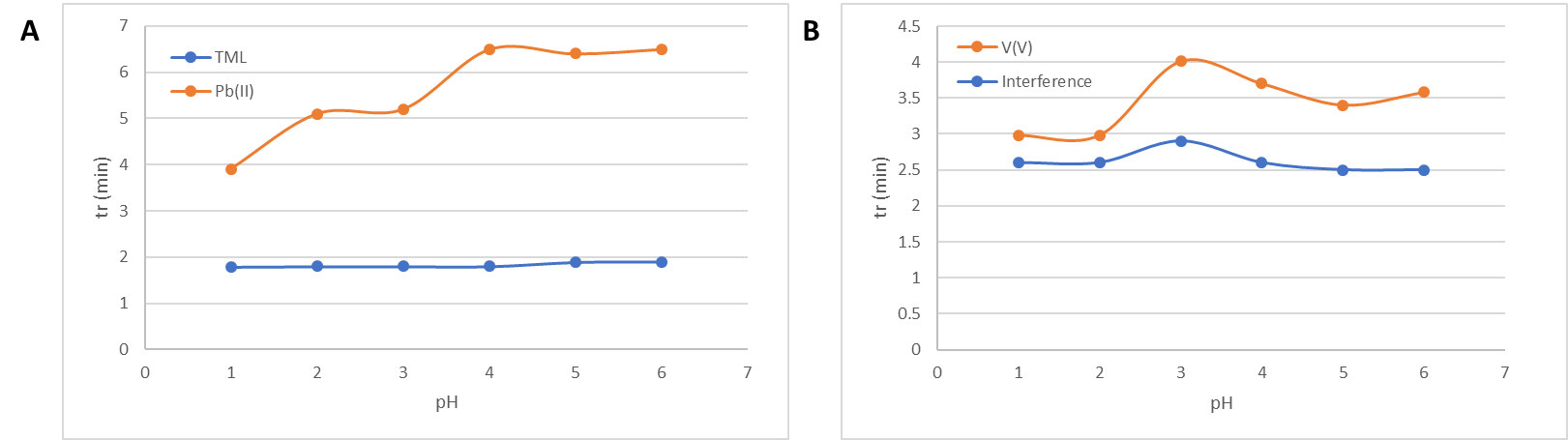


**Figure SM12** Optimization results of phase B pH for A) R1 (distance between retention times for TML and Pb^II^, and B) R2 (distance between retention times for V^V^ (and its interference).
